# Multiscale imaging reveals the presence of autophagic vacuoles in developing maize endosperm

**DOI:** 10.3389/fpls.2022.1082890

**Published:** 2023-01-06

**Authors:** Elsa Arcalís, Ulrike Hörmann-Dietrich, Eva Stöger

**Affiliations:** Institute of Plant Biotechnology and Cell Biology, Department of Applied Genetics and Cell Biology, University of Natural Resources and Life Sciences, Vienna, Austria

**Keywords:** multiscale imaging, storage vacuole, lytic vacuole, autophagy, maize endosperm

## Abstract

Cereal endosperm is solely devoted to the storage of proteins and starch that will be used by the embryo upon germination. The high degree of specialization of this tissue is reflected in its endomembrane system, in which ER derived protein bodies and protein storage vacuoles (PSVs) are of particular interest. In maize seeds, the main storage proteins are zeins, that form transport incompetent aggregates within the ER lumen and finally build protein bodies that bud from the ER. In contrast to the zeins, the maize globulins are not very abundant and the vacuolar storage compartment of maize endosperm is not fully described. Whereas in other cereals, including wheat and barley, the PSV serves as the main protein storage compartment, only small, globulin-containing PSVs have been identified in maize so far. We present here a multi-scale set of data, ranging from live-cell imaging to more sophisticated 3D electron microscopy techniques (SBF-SEM), that has allowed us to investigate in detail the vacuoles in maize endosperm cells, including a novel, autophagic vacuole that is present in early developmental stages.

## 1 Introduction

The seed endosperm is a triploid tissue that results from the fusion of the sperm nucleus with the two polar nuclei upon fertilization. Subsequently, the endosperm cell enters a phase of nuclear division without cytokinesis, that results in the nuclei arranging at the periphery of the central cell, which defines a large central vacuole. In several more rounds of nuclear division with cytokinesis and cell wall formation, the central vacuole fills up in a process known as cellularization (Becraft, 2012; Liu et al., 2022). From this moment on, the two major tissues in cereal endosperm differentiate: the outer aleurone and the starchy kernel endosperm. The aleurone layer is formed by polyhedral cells, arranged in a single layer in the case of maize, and contain lipids, proteins, vitamins and micronutrients. Together, the endosperm forms the main part of the seed and stores reserves in the form of starch and proteins that will support the embryo during germination (Baroux & Grossniklaus, 2019; Liu et al., 2022). It is interesting to note that aleurone and endosperm have very different fates despite their common origin. Thus, at maturity, the aleurone cells are the only living cells in the endosperm, and upon germination they synthesize and secrete hydrolases that mobilize the starch and proteins of the starchy endosperm. Otherwise, starchy endosperm cells undergo programmed cell death (PCD) during seed development and the starchy endosperm is dead at maturity. In this regard, the starchy endosperm is a very interesting tissue when it comes to protein synthesis, trafficking and storage, since it has reached a high degree of functional specialization to produce and store large amounts of proteins in a relative short period of time. Indeed, the endomembrane system of cereal endosperm is highly dynamic and undergoes rapid changes to synthesize seed storage proteins (SSPs) and store them in newly formed storage organelles (Arcalis et al., 2014)

In maize, 70% of the seed proteome is composed of storage proteins (Flint-Garcia et al., 2009), with zeins being the main storage proteins in maize endosperm. Zein synthesis in the endosperm starts as soon as 10 days after pollination (dap), increases steadily along development and reaches a peak around 25 dap (Arcalis et al., 2010). To cope with such a rapidly growing amount of protein, the biosynthetic machinery needs to expand and massive endoplasmic reticulum sheets are characteristic of maize endosperm around 15 dap (Arcalis et al., 2020). Zeins can be divided into 4 sub-families, and α-, ß-, γ- and δ-zeins are combined to form zein bodies with a highly conserved distribution pattern, such that alfa- and delta-zeins form the core of the zein body while gamma- and beta-zeins are located in its periphery. Under the electron microscope, the central core of zein bodies (alfa- and delta-zeins) typically shows a low electron density, surrounded by a peripheral belt (gamma- and delta-zeins) with higher electron density (Lending & Larkins, 1989).

Although zeins are the major storage protein in maize and ER derived zein bodies are by far the most abundant storage organelles, maize also synthesizes minor amounts of globulins that accumulate in protein storage vacuoles (Woo et al., 2001; Arcalis et al., 2010). Maize PSVs are already visible in early seed development as small compartments with electron dense inclusions. As development progresses, the PSVs increase in size and number until a stage close to maturity where their number drastically decreases (Arcalis et al., 2010). Additional to this study, there is relatively little information available on the PSVs of maize endosperm. In contrast, the vacuolar storage compartment in small grain cereals like wheat or barley has been well characterized. In both cases, large central vacuoles are present in the endosperm cells and act as final protein storage compartments for both globulins and prolamins, the latter being packaged in ER derived protein bodies (PBs) and transported to the vacuole bypassing the Golgi through an autophagy-like pathway (Levanony et al., 1992; Ibl et al., 2014) in which the involvement of *ATG* genes remains still unclear. In contrast, in seeds of different Arabidopsis *atg* mutants, a decrease of 12S globulins in favor of 12S globulin precursors compared to WT seeds was observed, pointing to a putative involvement of autophagy in SSPs delivery to the vacuole (Di Berardino et al., 2018). To date, such pathways have not been described in maize, at least not to a comparable extent, however, the reported morphological observations of vacuolar compartments at different stages of maize seed development are difficult to reconcile. Based on a combination of different microscopy techniques, in this study we therefore aim to further characterize the vacuolar compartments of the maize endosperm and clarify their role during seed development.

## 2 Materials and methods

### 2.1 Plant material

Maize wild-type (WT) maize plants (HiII) were grown in soil in a growth chamber with a 13-h photoperiod, 25/22°C Day/night temperatures and 70% relative humidity. Seeds were harvested at 15 and 21 days after pollination (dap), corresponding developmental stages 2 and 3 as described in Arcalis et al. (2010).

### 2.2 Confocal laser scanning microscopy

**Figure 1:**
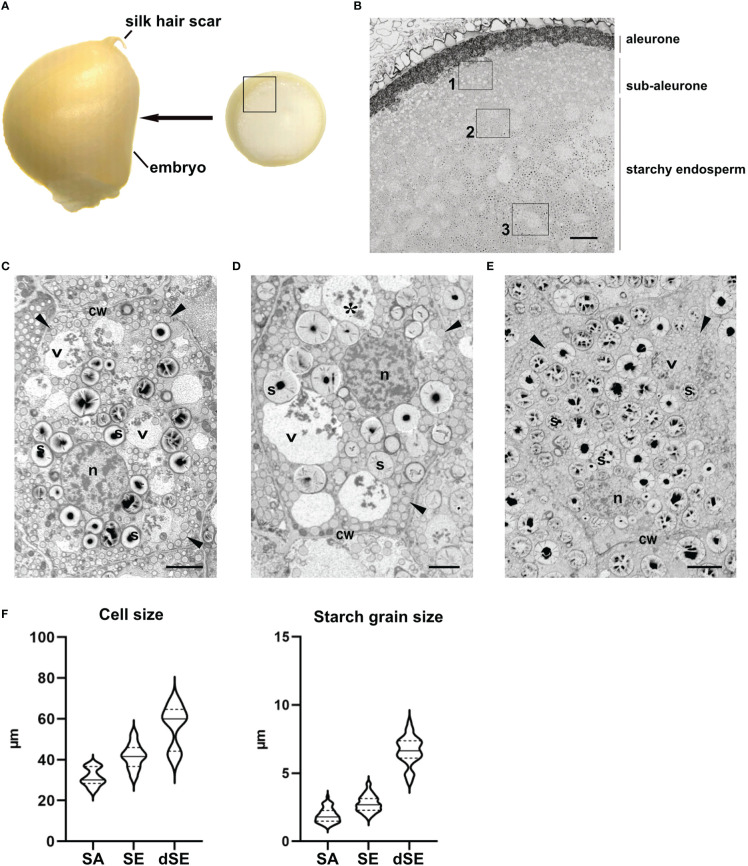
Mapping of the layers in the endosperm. **(A)** Thin slices of endosperm tissue were obtained immediately above the embryo (arrow). The inset corresponds to the area imaged in B. **(B)** SEM. Overview of the different endosperm layers. See the aleurone, sub-aleurone and starchy endosperm. A representative cell from the areas marked with the insets 1, 2 and 3 is depicted in C, D and E respectively. **(C-E)**. SEM. Sub-aleurone cell **(C)**, starchy endosperm cell **(D)**, deep-starchy endosperm cell **(E)**. Cell wall (cw), nucleus (n), starch (s), protein storage vacuole (*), zein bodies (arrowheads). **(F)** Cell and starch size grain in the different endosperm layers. Bars 100 µm **(B)**, 5 µm **(C, D)**, 10 µm **(E)**.

Seed cross sections were obtained with a razor blade from immediately underneath the silk hair (**Figure 1A**) and subjected to different stains prior to imaging under a Leica SP5 confocal laser scanning microscope (CLSM). PSVs were stained with neutral red 4 µM (Ibl et al., 2018; Stefano et al., 2018) and the ER was visualised by incubating the sections with 3 µM ERTracker green™ (Ibl et al., 2018). Monodansylcadaverine (MDC) was used to identify autophagic vacuoles as described in Contento et al. (2005). Briefly, seed sections were incubated in 50 µM MDC in PBS for 10 min and observed under the CLSM after several washes in PBS. Images produced by the Leica LAS software were processed using LASX (Leica Microsystems (UK) Ltd.), ImageJ (Rasband W., NIH, USA), Adobe Photoshop CS6 or Affinity Photo software.

### 2.3 Electron microscopy

#### 2.3.1 Sample preparation

Small tissue pieces including the aleurone were excised from seed cross sections obtained immediately underneath the silk hair (**Figure 1A**) and fixed and processed as described in Arcalis et al. (2020). Shortly, samples were fixed in 2.5% glutaraldehyde and 2% paraformaldehyde in 0.15 M cacodylate buffer overnight at 4°C. Subsequently, samples were washed with 0.15 M cacodylate buffer and transferred to 2% osmium tetroxide with ruthenium red for 1 h. After several washing steps with 0.15 M cacodylate buffer, tissue pieces were placed in 1% (w/v) freshly prepared thiocarbohydrazide solution for 45 minutes at RT and 2% aqueous osmium tetroxide for an additional hour. Samples were then washed in ultrapure water and immersed in uranyl acetate overnight. After washing with ultrapure water, the samples were transferred to Waltron’s lead aspartate (20 mM lead nitrate in a 30 mM L-aspartic acid solution) and washed with ultrapure water once again. Subsequently, the samples were dehydrated through an ethanol series, followed by dehydration in acetone. Tissue pieces were embedded in LV Resin and polymerized at 60°C for 48 h. For transmission electron microscopy (TEM), ultrathin sections were mounted on copper grids and observed in a FEI Tecnai G2 transmission electron microscope operating at 160 kV. For scanning electron microscopy (SEM), 200 nm sections were mounted on iridium tin oxide (ITO) glass. Once mounted on a SEM stub, sections were imaged in an Apreo SEM (Thermo Fisher Scientific, Thermo Scientific™), operating in Optiplan modus (2 kV, 0.1 nA).

#### 2.3.2 Serial block face imaging

Tissue blocks were trimmed and mounted as described in Arcalis et al. (2020). Image stacks were scaled and aligned with ImageJ and the segmentation was performed with Amira software (Thermo Fisher Scientific, United States) and the 3dmod graphic module of the IMOD software package (Boulder Laboratory of 3D Electron Microscopy of the Cell, University of Colorado at Boulder). At least five different stacks were obtained and one representative model was generated. At least five different stacks were obtained and one representative model was generated.

### 2.4 Size measurements

The cell size, as well as the size of starch grains in the sub-aleurone layer, the starchy endosperm and deep starchy endosperm were measured with Image J (n=10, for cell size measurements; n=50 for starch grains diameter). The values obtained were presented as violin plots, with median and quartiles indicated as lines.

To measure the size reduction of zein bodies present in the lumen of the vacuoles, the diameter of all zein bodies present in a vacuole was measured in 12 different images and compared to the diameter of the zein bodies in the cytoplasm, where n is the number of the zein bodies in the vacuole and 2n is the number of zein bodies measured in the cytoplasm.

## 3 Results

In previous studies, we have followed the maturation of maize endosperm using a time series. Thus, we defined three developmental stages based on cell morphology rather than days after pollination: stage 1 (~10-14 daps), stage 2 (~15-20 daps) and stage 3 (~21-30 daps) (Arcalis et al., 2010). During maturation, there is an age gradient in the cereal endosperm towards the center of the seed. The so-called centripetal maturation of the cereal endosperm is advantageous for time course studies as developmental changes at cell level can be easily followed within the same sample. The results presented in this work show cells in different layers of the endosperm in seed developmental stage 2, obtained from cross sections immediately under the silk hair scar (**Figure 1A**). In order to keep the data consistent, we mapped the maize endosperm cross-sections to define the different endosperm layers (**Figures 1B-E**). At this developmental stage, the peripherical aleurone, consisting of polyhedral cells that have a high electron density due to their high lipid content, forms a monolayer. Immediately below is the sub-aleurone, consisting of a few layers of young cells that contain abundant electron-transparent structures, followed by the core starchy endosperm, which constitutes the bulk of the seed (**Figure 1B**). Based on cell size and starch grain size, we have defined three layers within the starchy endosperm: subaleurone (SA, **Figure 1C**), starchy endosperm (SE, **Figure 1D**) and deep starchy endosperm (dSE, **Figure 1E**). Cells in the subaleurone are the smallest (31,42 ± 4,13 µm), and cell size progressively increases toward the center of the seed (41,77 ± 5,70 and 56,73 ± 9,72 µm in the starchy endosperm and deep starchy endosperm, respectively). The size of the starch grains also increases as cells mature and we have established this as the reference parameter for classifying a cell within the endosperm. Starch grains in the subaleurone cells have an average diameter of 1,91 ± 0,52 µm, in the starchy endosperm the diameter is 2,76 ± 0,59 µm, and in the deep starchy endosperm they reach an average of 6,65 ± 1,03 µm (**Figure 1F**). In some sections, we were able to measure starch grains of more than 10 µm in deeper layers of the starchy endosperm (*very deep starchy endosperm*).

### 3.1 Vacuoles in maize endosperm cells along development

**Figure 2:**
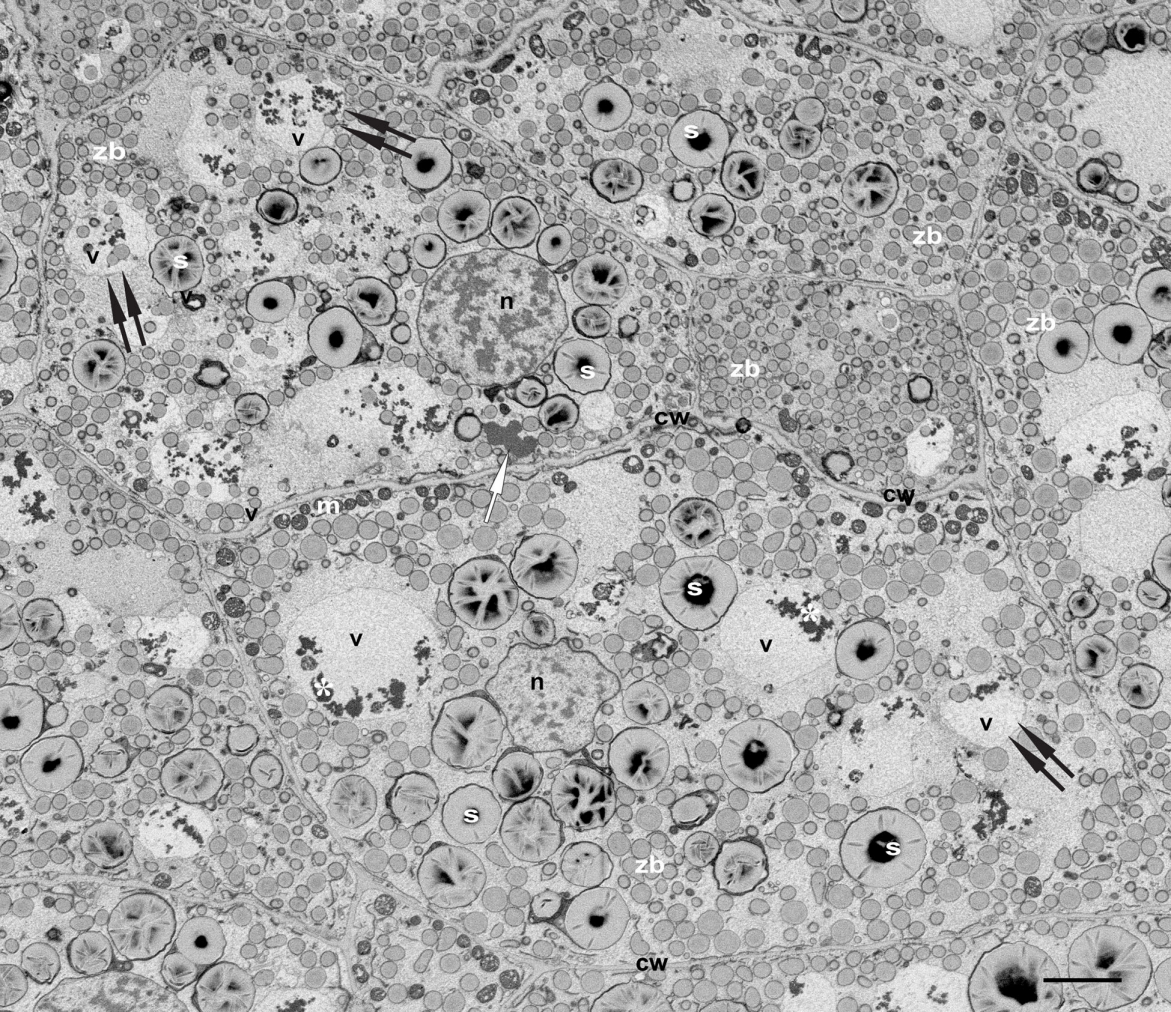
Vacuoles in developing maize seeds. SEM. Developmental stage 2, overview starchy endosperm. Cells contain 4-5 turgent vacuoles (v), that include electrondense globulin inclusions (*). Note the presence of zein bodies in some of them (double arrows). See a single protein storage vacuole (white arrow). Bar 5 µm.

**Figure 3:**
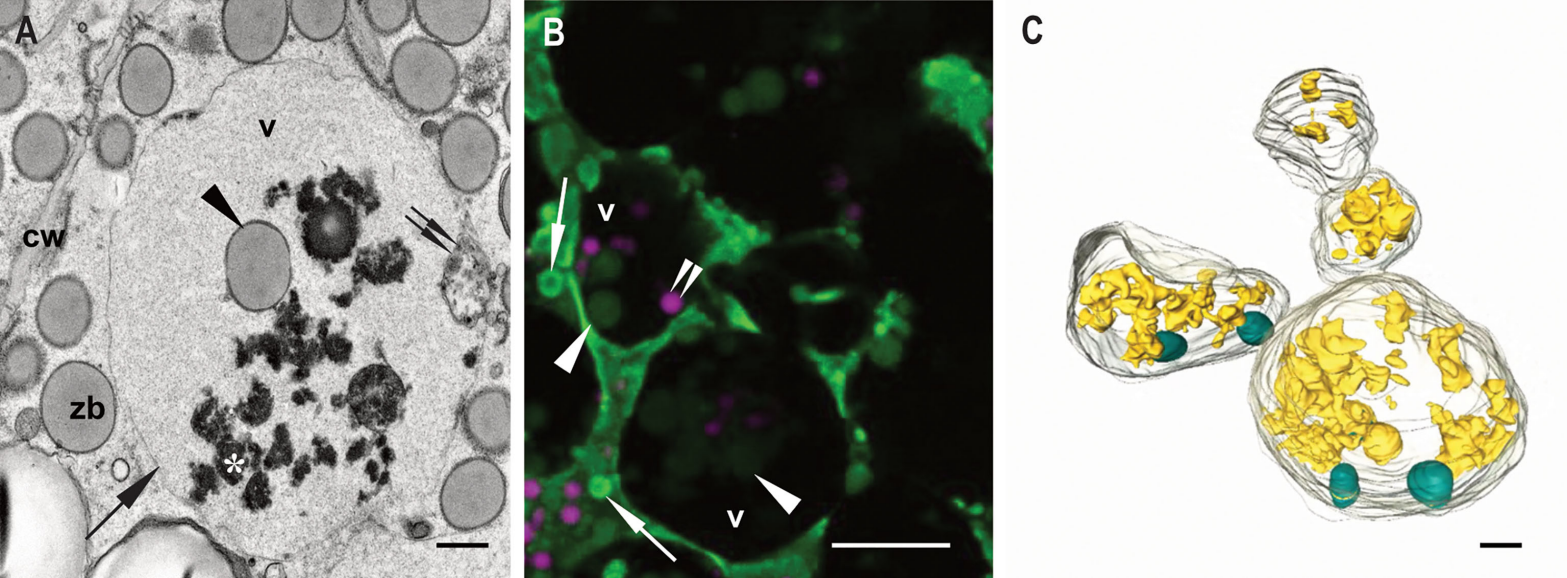
Zein bodies in endosperm cell vacuoles. Developmental stage 2. Starchy endosperm. **(A)** TEM. Vacuole (v) containing a zein body (arrowhead), globulin inclusions (*) and also some membrane structures (double arrow). Tonoplast (arrow). **(B)** CLSM, WT seed stained with ER Tracker™ green and neutral red. Several zein bodies (arrowheads) together with globulin inclusions (double arrowheads) within a vacuole (v). White arrows mark zein bodies within the cytoplasma. **(C)** SBF-SEM. 3D rendering of vacuoles. Zein bodies (green) and globulin inclusions (yellow) within a vacuole. Bars 1 µm **(C)**, 10 µm **(B)**, 1 µm **(C)**.

**Figure 4:**
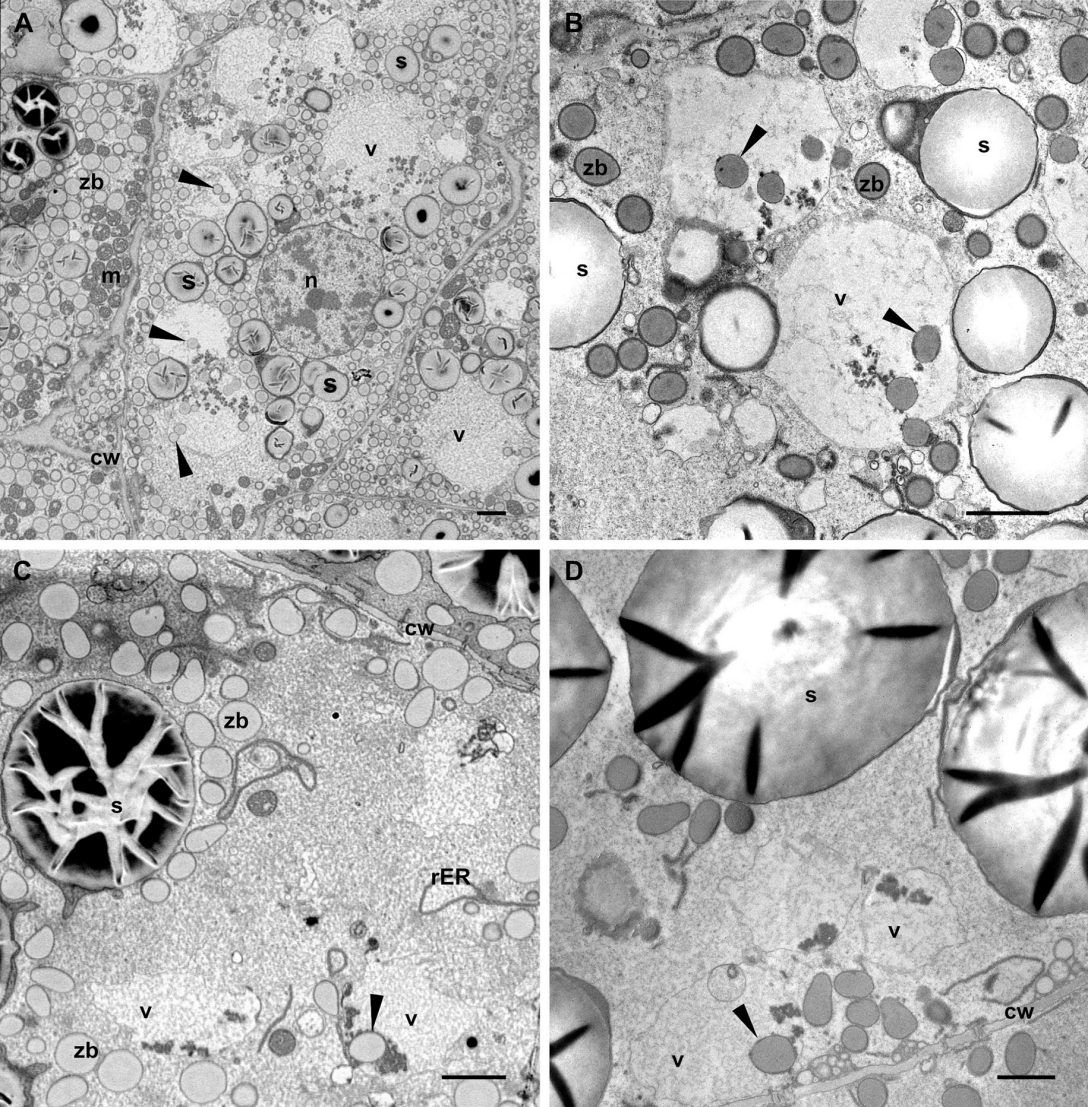
Vacuoles in different endosperm layers. Developmental stage 2. TEM. **(A)** Subaleurone, **(B)** Starchy endosperm, **(C)** Deep starchy endosperm, **(D)** Very deep starchy endosperm (see legend in **Figure 1**). Note the presence of zein bodies in the vacuoles (arrowheads) in all layers of the endosperm. Cell wall (cw), mitochondria (m), rER (rough endoplasmic reticulum), starch (s), zein bodies (zb). Bars 2 µm.

Our main target in the present study are the vacuoles present in developing maize endosperm cells. Seed storage vacuoles, containing globulin-type storage proteins, generally appear as electron-dense organelles under the electron microscope, making them easy to identify in both cereals (Tian et al., 2018) and dicot seeds (Feeney et al., 2018). In the case of maize endosperm, PSVs containing corn legumin-1 and corn alfa-globulin have been previously described (Woo et al., 2001; Arcalis et al., 2010; Reyes et al., 2011). We have found PSV-like structures in maize starchy endosperm cells in developmental stage 2, as high electron dense, amorphous structures, that are rather scarce (**Figure 2**). However, these storage vacuoles become more abundant as seed development progresses, and at 21 daps (stage 3), endosperm cells present multiple vacuoles with globulin inclusions (**Supplementary Figure 1**). In addition to these globulin-containing storage vacuoles, we could identify a second vacuolar compartment in young maize endosperm. Indeed, at seed developmental stage 2 (approx.15 dap), the most relevant vacuolar compartments in sub-aleurone cells are spheroidal vacuoles of around 4 µm in size (**Figure 2**). Interestingly, these vacuoles seem to be restricted to this developmental stage, as they are not present in younger seeds or in later stages of seed development (Arcalis et al., 2010, **Supplementary Figure 1**). Vacuoles in endosperm cells at developmental stage 2 contain loose globulin-like electron dense aggregates that, unlike in the above described PSVs, do not completely fill the lumen (**Figure 2**, **3A**). Globulins are not the only content of these vacuoles, and membraneous material and, surprisingly, zein bodies are also found within their lumen (**Figures 2**, **3A**, **4**). In order to further characterize this compartment, we combined conventional electron microscopy with live cell imaging and three-dimensional EM techniques. The use of an ER membrane stain for live cell imaging and the high resolution of transmission electron microscopy reveal that zein bodies within the vacuole have lost the ER membrane surrounding the zein bodies in the cytoplasm (**Figures 3A, B**). Thus, upon ER Tracker™ staining, the zein bodies in the cytoplasm appear surrounded by a green labelled membrane, while those within the vacuole are lacking a membrane and can only be observed because of their autofluorescence (**Figure 3**). Similarly, under the transmission electron microscope, zein bodies without surrounding ER membrane, could be observed within a vacuole (**Figure 4B**, **Supplementary Figure 2**). Further, we used Serial Block Face-SEM to generate models that allow the 3D reconstruction of these vacuoles and show that the zein bodies are enclosed in the vacuolar lumen, excluding any kind of tonoplast invagination that could be misleading when observed with conventional 2D electron microscopy (**Figure 1** and **Supplemental Video 1**).

**Figure 5:**
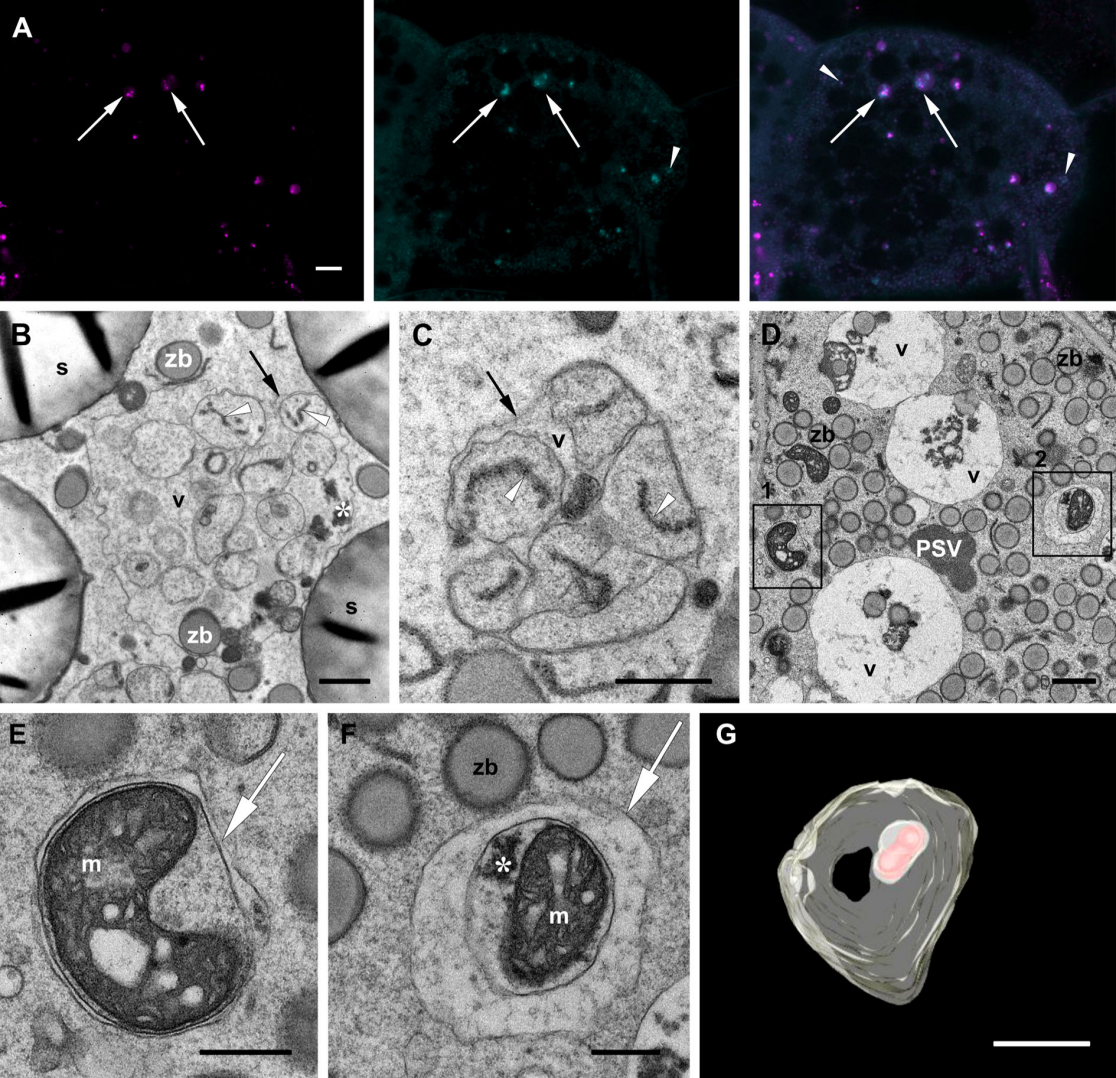
Autophagic vacuoles. Developmental stage 2. Starchy endosperm. **(A)** CLSM. Neutral red (magenta), MDC (cyan), merged. Vacuoles containing autophagosomes (arrows), autophagosomes within the cytoplasma (arrowheads). **(B-F)**. TEM. Examples of ERphagy **(B, C)**. See the ER fragments (white arrowhead) included in autophagic bodies within the vacuole (v). Examples of mitophagy **(D-F)**. **(E)** and **(F)** correspond to the enlargements of insets 1 and 2 respectively found in **(D)**. **(E-F)**. Autophagosomes (arrow) enclosing a mitochondrion (m). **(G)** SBF-SEM. 3D rendering of vacuoles. See an autophagic body containing a mitochondrion within a vacuole (pale yellow, vacuolar membrane; red, mitochondiron). Globulins (*), protein storage vacuole (PSV), tonoplast (black arrow), zein bodies (zb). Bars 10 µm **(A)**, 1 µm **(B-G)**.

Vacuoles containing zein bodies and also small amounts of globulins, as well as various membrane structures, were found in all layers of the endosperm at stage 2, from the young cells of the sub-aleurone to the older ones in the starchy endosperm layers (**Figure 4**). While the vacuoles in the sub-aleurone cells appear turgent and therefore mostly spheroidal (**Figures 4A, B**), the vacuoles in the deeper layers of the endosperm are less abundant, have lost the turgor and appear slightly smaller (**Figures 4C, D**, **1E**). For the zein bodies, a higher number per vacuole was observed in early developmental stages (up to 9 zein bodies in one vacuole in the sub-aleurone, **Figure 4A**), whereas toward the center of the seed, where cells are in more advanced developmental stages, this number decreases (**Figures 4C, D**). It is also important to note that compared to the zein bodies in the cytoplasm, the zein bodies in the vacuole have lost not only the ER membrane mentioned earlier, but also the peripherical dark electron-dense ring corresponding to gamma- and beta-zeins, and are therefore slightly smaller (**Figures 4B**, **5A**). Indeed, we measured the diameter of zein bodies in vacuoles and confirmed that their diameter is 30% smaller compared to zein bodies in the cytoplasm (**Supplementary Figure 2**).

### 3.2 Autophagic vacuoles in maize endosperm

The apparent partial digestion of the zein bodies within vacuoles in young endosperm cells calls into question the storage role of such vacuoles and is more indicative of a lytic function. To further corroborate their role as autophagic vacuoles, we used monodansylcadaverine (MDC), a convenient acidotropic dye that stains autophagosomes and autophagic bodies (Contento et al., 2005; Pu & Bassham, 2016). Indeed, autophagosome-like structures could be identified within the vacuoles as bright punctae together with the globulin inclusions also present in the vacuolar lumen. Additionally, few fluorescent punctae could be observed in the cytoplasm (**Figure 5A**). Thus, the use of MDC confirmed that the numerous vacuoles observed in the different layers of the endosperm are involved in autophagic processes. Our observations under the electron microscope show that, in addition to the zein bodies and globulin inclusions already mentioned, several autophagic bodies with different contents are indeed found within the vacuoles in the starchy endosperm (**Figures 5B–D**). Most interesting are the autophagic bodies containing ER fragments, clearly indicating ERphagy (**Figures 5B, C**). Examples of mitophagy are also readily found in the cytoplasm, as seen in **Figures 5D–F**, where two autophagosomes containing mitochondria with malformed cristae are depicted. A 3D model of an autophagic body within a vacuole containing a mitochondrion is shown in **Figure 5G** and in **Supplementary Video 2**.

**Figure 6:**
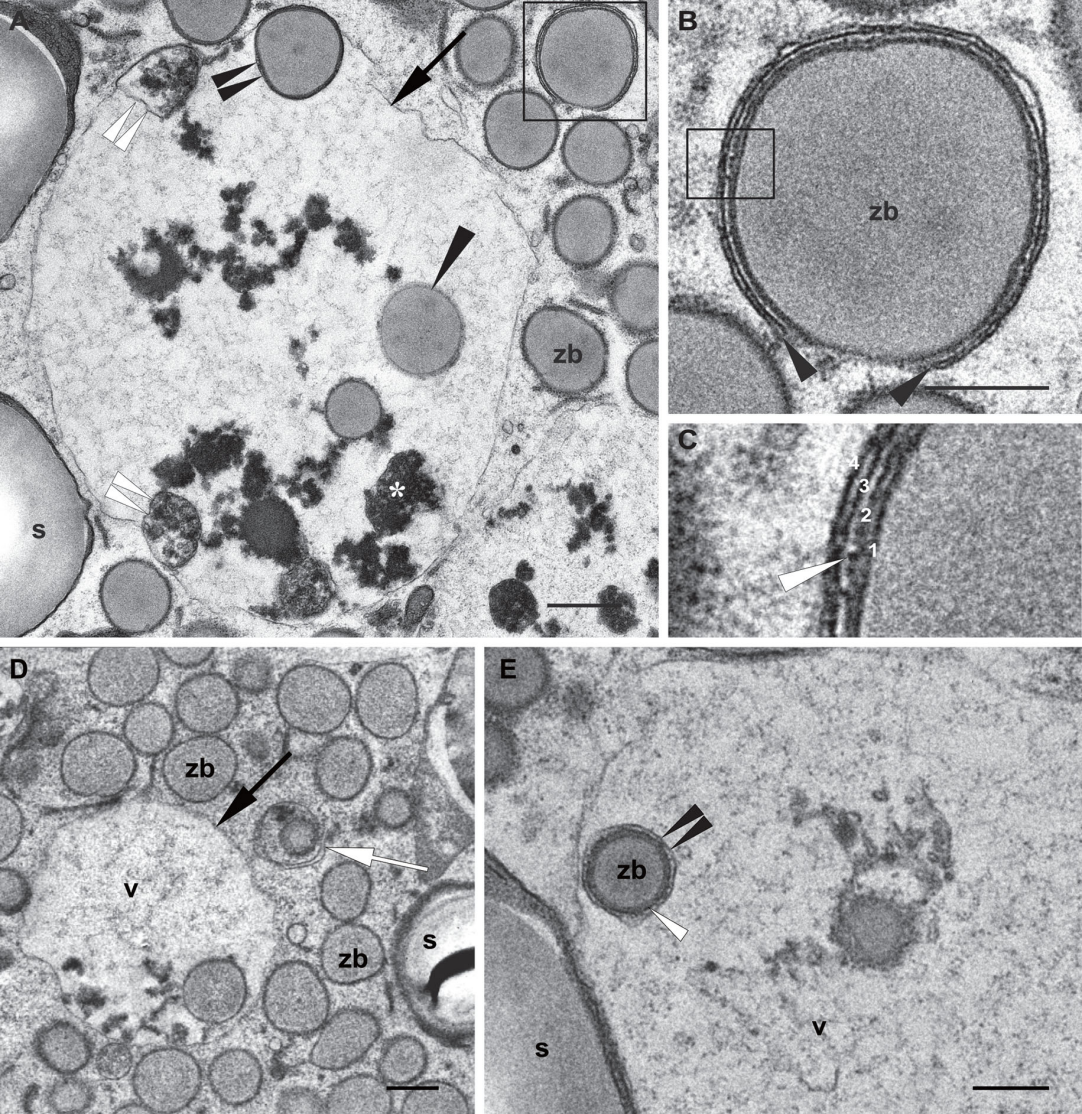
Autophagic pathway into the vacuole. Developmental stage 2, starchy endosperm. TEM. **(A)** Vacuole (v) containing globulins (*), membrane structures (double white arrowheads) and zein bodies. Note a just incorporated zein body, still preserving a membrane (double black arrowheads) and a zein body without any membrane (arrowhead) in the vacuolar lumen. The inset is enlarged in **(B)**. **(B)** A phagophore surrounding a zein body (arrowheads). **(C)** Enlargement of the inset in **(B)** 1. Edge of the zein body, 2. Protein body membrane, ER derived, see a ribosome (white arrow), 3, 4. Phagophore membranes. **(D)** Autophagosome including a zein body and an electrondense globulin deposit (arrow). **(E)** Zein body within a vacuole, still surrounded by an ER membrane (white arrowhead) and an autophagosomal single membrane (double arrowheads). Starch (s), vacuole (v), zein body (zb). Bars 0,5 µm.

Next, we observed under the electron microscope autophagic-like structures within the cytoplasm of the starchy endosperm cells as small, typically cup-shaped phagophores (**Figures 6A, B**). Following autophagosome biogenesis, isolation membranes expand and seal to complete the autophagosome formation (**Figure 6**). These autophagosomes usually include a single zein body (**Figures 6B–D**) and may in addition also include electron dense globulin-like material (**Figure 6D**). The same is observed in **Figure 5F**, showing an autophagosome containing a mitochondrion together with a globulin inclusion. After fusion with the tonoplast, autophagic bodies are found in the vacuolar lumen (**Figure 6E**). The remaining autophagosomal membrane is subsequently digested and releases its content into the vacuolar lumen, as mentioned earlier. It is important to note that in the case of zein bodies contained in autophagic bodies, the ER membrane surrounding the protein body is still present (**Figure 6E**), indicating that it is digested within the vacuole and not before, further highlighting the lytic nature of this vacuole.

## 4 Discussion

The results and conclusions presented in this study are mainly based on microscopic data, with particular emphasis on ultrastructural investigation. Our study is therefore an example of the combined use of a wide range of currently available microscopy techniques that are ideally suited to address questions in plant cell biology at the single cell level and at the highest resolution. For example, the use of different fluorescent dyes in live-cell imaging and the ability to work with large fields of view allowed us to rapidly identify vacuolar compartments in the bulk of the endosperm. However, conventional live-cell imaging is limited by its resolution (200 nm in the x-y plane) and also by the fact that only fluorescent signals can be detected while unlabeled molecules or organelles cannot be visualized with sufficient quality (Weiner et al., 2022). The superior resolution of electron microscopy plus the use of heavy-metals in sample preparation that contrast the entire cell, allowing the ultrastructure of the whole cell to be examined at once (Martell et al., 2017), makes electron microscopy the perfect complement to live-cell imaging and enables structural research across the scales.

Indeed, we have previously published several images of cereal endosperm produced with transmission electron microscopy (Arcalis et al., 2014; Arcalis et al., 2020) Here, we make use of backscattered electrons to generate 2D images of embedded tissue sections using scanning electron microscopy (SEM). In parallel with live-cell imaging, SEM imaging of tissue sections allows the imaging of large tissue sections, combined with the advantage of higher resolution (see **Figure 2A** and **Supplementary Figure 1A**, where images with 75 and 700 µm horizontal fields of view are shown). In addition, it circumvents another problem with live-cell imaging in developing maize endosperm, which arises because the ratio of injured tissue to intact tissue is very high as a result of sectioning and the imaging time of fresh tissue is rather limited. By visualizing the entire tissue at high resolution, we were able to easily assign our observations at higher magnification (TEM) to specific cell layers within the endosperm, allowing a comprehensive mapping of the vacuolar compartments of developing maize seeds in different cell layers and thus different cell developmental stages. The images and techniques discussed to this point, lack three-dimensional information at high resolution. However, the development of sophisticated 3D imaging techniques in recent years has advanced ultrastructural research. For example, Ding et al. (2022) published a comprehensive multiscale study on autophagy pathways in developing maize aleurone cells, combining electron microscopy, live cell imaging and electron tomography. Electron tomography provides 3D information of small cell volumes at TEM resolution. In contrast, serial block face-SEM imaging offers the possibility to investigate larger volumes while mantaining a high level of detail. The 3D models shown in the present work confirm the full incorporation of zein bodies and other cargo into the vacuolar compartment, providing additional information to conventional 2D images.

By combining the microscopic tools described above, we were able to establish that the presence of vacuoles with different morphological and funtional characteristics in developing maize endosperm cells follows a distinct pattern. Thus, younger cells in the peripheral layers of starchy endosperm at developmental stage 2 contain abundant vacuoles to which we could assign features of a lytic vacuole as we found that they are involved in the uptake and degradation of cytoplasmic components including mitochondria, ER segments, and protein bodies. These vacuoles decrease in size and number towards the center of the seed, as cells grow older and are already less significant in the deep starchy endosperm at developmental stage 2 and later in seed development they were absent already in outer endosperm layers. At the same time, we observed that the globulin-filled PSVs, which were almost anecdotal in younger cells, increased in importance during development and became the major vacuoles in endosperm cells from 20 dap onwards. While the role of these latter PSVs in cereal endosperm in general and in maize in particular is clear, as they are storage organelles in a tissue devoted to the storage of nutrients for the embryo, the presence of a lytic compartment in a storage tissue is puzzling. By using MDC, we could identify punctate signals likely corresponding to autophagosomal structures in these vacuoles, indicating their involvement in autophagic processes. This is consistent with earlier observations of phagophore- and/or autophagosome-like structures in 18 dap old peripheral endosperm cells of maize plants expressing a reporter construct containing YFP fused to ATG8a (Li et al., 2015). These autophagic bodies as well as the ones described here were particularly evident in the younger endosperm cells, where it is known that the synthesis and accumulation of storage proteins is most pronounced in cereal endosperm (Shewry et al., 2020). The onset of storage protein synthesis is accompanied by elevated levels of BiP, suggesting that the synthesis of storage proteins, while being physiological, challenges the protein folding capacity of the ER (Muench et al., 1997; Wakasa et al., 2011). Additionally, a direct correlation between storage protein synthesis and elevated unfolded protein response (UPR) signalling was observed during the early stages of seed development in *Brachypodium distachyon*, suggesting that meeting protein synthesis demands during endosperm development puts a strain on the ER and seed storage protein accumulation needs to be balanced in different physiological states as far as possible unless or until overwhelmed by massive storage protein synthesis or structural defects (Kim et al., 2017) (Vitale & Pedrazzini, 2022). One of the options to mitigate ER stress is the delivery of proteins to hydrolytic compartments *via* autophagy (van Anken et al., 2021). Zhang et al. (2020) described an ER stress- related autophagy in maize aleurone cells. While zeins are also expressed in maize aleurone cells (Reyes et al., 2011), the bulk of zein synthesis occurs in the endosperm and therefore, the evident cases of ERphagy we observed in developing maize endosperm cells may also be linked to ER stress induced by zein synthesis. In this respect, our observations of autophagosomes containing mitochondria could eventually be a consequence of imbalanced cellular stress, and an example of selective autophagy (Su et al., 2020) removing damaged cell components, and this is supported by the dilated cristae of the observed mitochondria. Autophagy of whole zein bodies is more difficult to explain, but could perhaps be considered a side effect of ERphagy during endosperm development. In general, the delivery of prolamin bodies to vacuolar storage compartments in cereal endosperm cells is well known and has been described as an autophagy-like process, particularly in wheat and barley (Ibl et al., 2014; Michaeli et al., 2014). More recently, the delivery of zeins to the protein storage vacuoles in maize aleurone was identified as microautophagy that does not involve the ATG8/ATG12 conjugation system or assembly of autophagosomes (Ding et al., 2022). However, the delivery of zein bodies into a lytic vacuole *via* macroautophagy observed in the present study represents a different case. Indeed, the subsequent reduction of protein body size in the vacuoles emphasizes their lytic character, which raises questions, however, because a storage organelle such as a zein body is not normally expected to be degraded in developing seeds. On the other hand, ectopic prolamin bodies induced by zeolin, a zein-derived chimeric storage protein, were reported to induce ER stress and were subject to Atg8-mediated autophagy and vacuolar delivery in leaf protoplasts (Yang et al., 2016) Also in cereal endosperm, a proportion of newly synthesized storage protein is targeted for degradation, suggesting that some of the proteins fail to fold properly (Ohta and Takaiwa, 2015, Cao et al., 2022). A recent, comprehensive quantitative analysis indicated that as much as 25% of nascent seed storage proteins undergo degradation in early stages of developing wheat endosperm (Cao et al., 2022; Vitale & Pedrazzini, 2022). Thus, the vacuolar degradation of zein bodies and most probably also globulins in young maize endosperm cells may reflect part of the process involved in the turn over of storage proteins that occurs in developing cereal endosperm despite the considerable energy costs associated with such a cycle of synthesis and degradation (Vitale and Pedrazzini, 2022).

Here, we have produced valuable information about the structure of developing maize endosperm and therefore provide a unique example of a multiscale imaging approach to address cell biology questions, based solely in microscopy. Further development of imaging techniques will enable more complete studies across scales, in plant cell science in general and in seed biology in particular.

## Data availability statement

The original contributions presented in the study are included in the article/**Supplementary Material**. Further inquiries can be directed to the corresponding author.

## Author contributions

EA and ES contributed to the conception and design of the study and wrote the manuscript. EA and UH-D designed and carried out the electron microscopy and SBF-SEM experiments. EA analyzed the data and generated the 3D models. All authors contributed to the article and approved the submitted version.
