# Supplementary material for: Multiscale imaging reveals the presence of autophagic vacuoles in developing maize endosperm
Source: Front Plant Sci. 2023 Jan 6;13:1082890. doi: 10.3389/fpls.2022.1082890 (PMC9853038; doi:10.3389/fpls.2022.1082890)
Supplement: Supplementary file 1 [file DataSheet_1.docx]

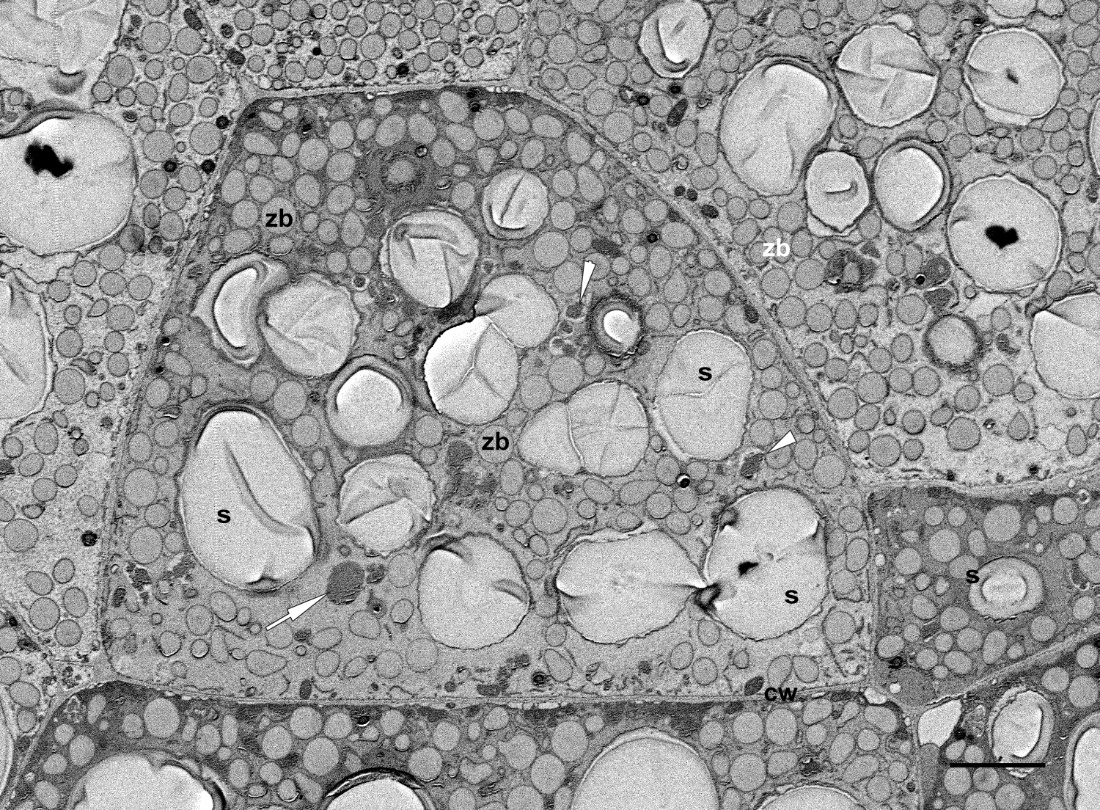


**Supplemental Figure 1.** SEM. Developmental stage 3, overview starchy endosperm. Several vacuoles containing large globulin inclusions are present (white arrows). Cell wall (cw), mitochondria (m), nucleus (n), starch (s), zein bodies (zb). Bars 5 µm.


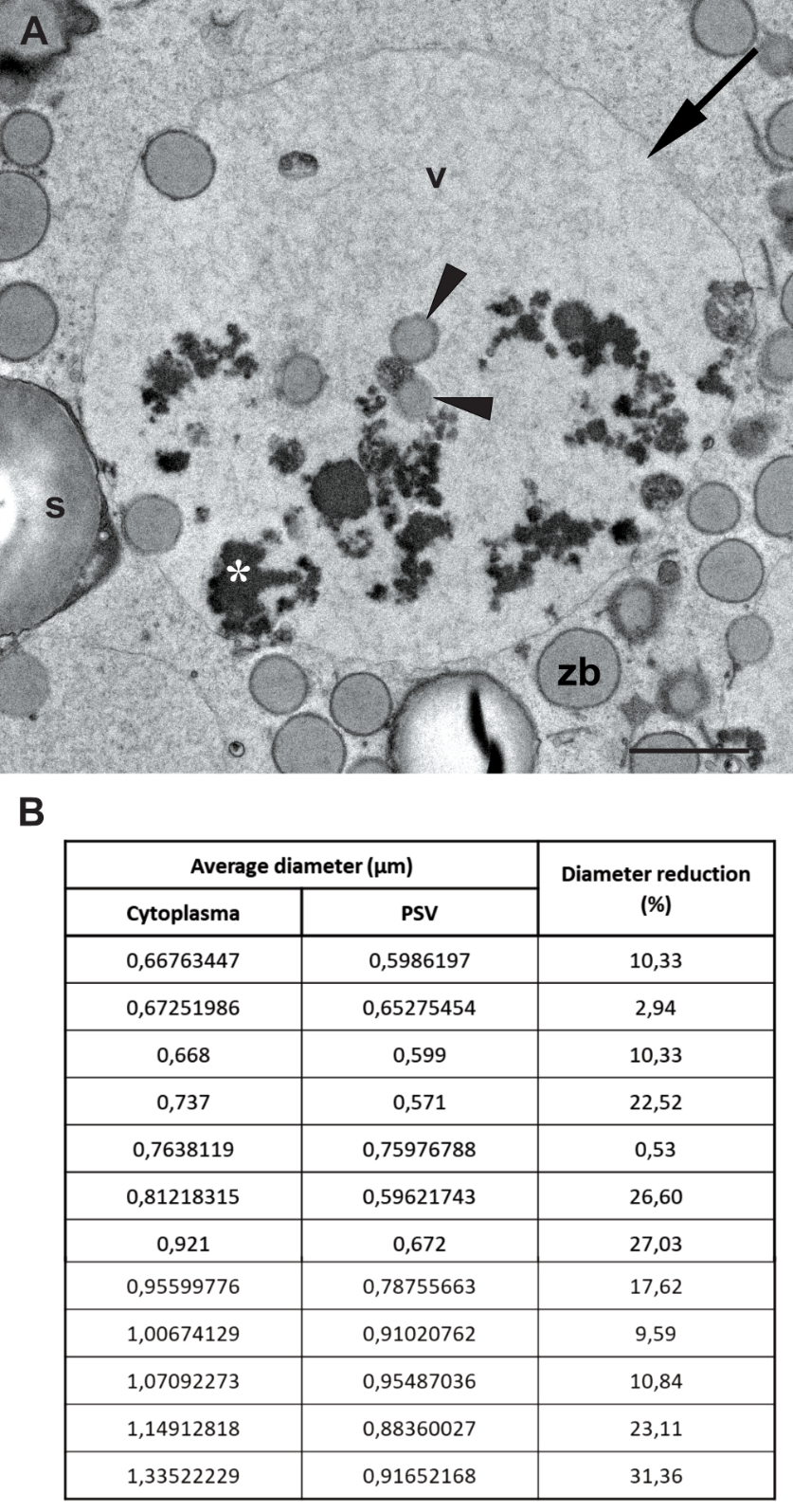


**Supplemental Figure 2. Diameter reduction of the zein bodies within a vacuole.**

A.TEM. Note the significant reduction of the diameter of the zein bodies in the vacuolar lumen (arrowheads), compared to that of those in the cytoplasm (zb). Globulins (*), starch (s), tonoplast (arrow), vacuole (v). Bar 2 µm. B. Measurements of zein body diameter in 12 different pictures.
